# Compulsory admission at first presentation to services for psychosis: does ethnicity still matter? Findings from two population-based studies of first episode psychosis

**DOI:** 10.1007/s00127-019-01685-y

**Published:** 2019-03-20

**Authors:** Sherifat Oduola, Tom K. J. Craig, Jayati Das-Munshi, Francois Bourque, Charlotte Gayer-Anderson, Craig Morgan

**Affiliations:** 1 School of Health Sciences, University of East Anglia, Norwich Research Park, Norwich, NR4 7TJ UK; 2 Department of Health Service and Population Research, Institute of Psychiatry, Psychology & Neuroscience, King’s College London, De Crespigny Park, Denmark Hill, London, SE5 8AF UK; 3 Division of Social and Cultural Psychiatry, Douglas Mental Health University Institute, McGill University, Montreal, QC H4H 1R3 Canada; 4 South London & Maudsley NHS Foundation Trust, Denmark Hill, London, SE5 8AZ UK

**Keywords:** Compulsory admission, Pathways to care, Ethnicity, Psychosis, African, Caribbean, First episode psychosis

## Abstract

**Objectives:**

Compared with the majority population, those from minority ethnic groups in the UK are more likely to be admitted compulsorily during a first episode of psychosis (FEP). We investigated whether these disparities in pathways in to care continue.

**Methods:**

We analysed data from two first episode psychosis studies, conducted in the same geographical area in south London 15 years apart: the Aetiology and Ethnicity in Schizophrenia and Other Psychosis (AESOP) and the Clinical Record Interactive Search-First Episode Psychosis (CRIS-FEP) studies. The inclusion/exclusion criteria for case ascertainment for first episode psychosis were identical across the two studies. We performed multivariable logistic regression to estimate odds of compulsory admission by ethnic group, controlling for confounders.

**Participants:**

Two hundred sixty-six patients with first episode psychosis, aged 18–64 years, who presented to mental health services in south London in 1997–1999 and 446 with FEP who presented in 2010–2012.

**Results:**

When the two samples  were compared, ethnic differences in compulsory admission appear to have remained the same for black African patients, i.e. three times higher than white British in both samples: AESOP (adj. OR = 3.96; 95% CI = 1.80–8.71) vs. CRIS-FEP (adj. OR = 3.12; 95% CI = 1.52–6.35). Black Caribbean patients were three times more likely to be compulsorily admitted in AESOP (adj. OR = 3.20; 95% CI = 1.56–6.54). This was lower in the CRIS-FEP sample (adj. OR = 1.68; 95% CI = 0.71–3.98) and did not meet conventional levels for statistical significance.

**Conclusion:**

Ethnicity is strongly associated with compulsory admissions at first presentation for psychosis with evidence of heterogeneity across groups, which deserves further research.

## Introduction

With the passage of time, some ethnic minority groups and their succeeding generations are more integrated into UK society than others. For example, the peak of migration to the UK in some ethnic minority groups was more than 70 years ago, while for others it was as recent as a decade ago [1, 2]. This is an important social change which may shed light on how people engage with health services. Furthermore, mental health service provision has changed in recent times, psychiatric hospital beds have substantially reduced [3, 4] and new services such as early intervention for psychosis in the community have been introduced [5]. Given these changes, it is unclear to what extent ethnicity is still associated with compulsory admissions during first episode psychosis.

Several studies have shown that compulsory admissions during a first episode psychosis are more likely among minority ethnic group patients [6–8], relative to white British patients. This evidence is particularly strong for the black ethnic group patients, with odds ratios ranging from twofold to fourfold [6, 9]. In some studies, investigators have attributed the disparities to differences in clinical presentations and have suggested that black patients are more severely disturbed [10, 11], or have poorer insight [11, 12] at presentation, which necessitate compulsory admission. However, it seems unlikely that differences in clinical presentation can alone account for the observed differences, as a number of studies in which clinical characteristics, such as diagnosis and mode of illness onset, were adjusted for still showed higher odds of compulsory admission [8, 13]. Other studies have reported discrimination, immigration status, and racial stereotyping as possible contributors to observed differences [14, 15]. The higher rates of compulsory admission among black and minority ethnic groups remain a major concern for patients, mental healthcare providers and policy makers [14]. So much so, in the UK for example, the government published a report on reducing the inequality in detention rates in the white paper ‘Delivering Race Equality in Mental Health Care’ [16].

A recent interim report from the Independent Review of the Mental Health Act, commissioned by the UK government, highlighted the disparities in compulsory admissions for black African and black Caribbean patients [17]. Studies have shown that, compared with white British patients, black African patients were four to five times, and black Caribbean patients were around two times more likely to be compulsorily admitted to hospital at first presentation for psychosis [7, 13, 18]. This suggests that black African patients may be at even higher risk of being compulsorily detained, but little is known about the associated factors.

We sought, then, to investigate the question of whether ethnic differences in compulsory admission during first episode psychosis have reduced over time. We used data from two population-based studies of first episode psychosis, namely the Aetiology and Ethnicity in Schizophrenia and Other Psychosis (AESOP, 1997–1999) and the Clinical Record Interactive Search-First Episode Psychosis (CRIS-FEP, 2010–2012) carried out in the same catchment areas, to assess for differences over time. Given the government strategies [16, 17] on reducing the inequalities in involuntary admissions, implemented after the AESOP study, we tested the hypothesis that, compared with 15 years ago, the differences in the odds of compulsory admissions at first presentation to services for psychosis by ethnicity would be reduced.

## Methods

### Samples

The AESOP and CRIS-FEP studies were conducted within the same inner city areas in London, UK. The areas covered were the London boroughs of Lambeth (total population 303,086) and Southwark (total population 288,283) (ONS, 2011a), served by the South London and Maudsley NHS Foundation Trust (SLaM). The two samples were drawn using identical inclusion and exclusion criteria and measurements of key variables, i.e. compulsory admission and sociodemographic characteristics.

### Study design, setting and participants

The methods used in AESOP have been widely published elsewhere [7, 19]. Briefly, AESOP is a three-centre study conducted in south London, Nottingham, and Bristol, in which cases with a first episode of any psychotic disorder (i.e. ICD F20-29, F30-33) were identified through liaison between the study team and mental health services in the study catchment areas. In this report, we included only cases from the AESOP London site (Lambeth and Southwark).

The CRIS-FEP study is a case register study of cases with a first episode of any psychotic disorder (i.e. ICD F20-29, F30-33). All patients presenting to the South London and Maudsley NHS Trust adult mental health services in Lambeth and Southwark for the first time with a psychotic disorder (including F20-29 and F30-33 in the ICD 10) between May 2010 and April 2012 were identified using the South London and Maudsley NHS Trust (SLaM) Clinical Records Interactive Search (CRIS) system [20], which provides fully anonymised access to all SLaM electronic clinical records. We used the structured language query [21, 22] to interrogate and extract information in CRIS free-text fields, based on our inclusion criteria, i.e. we defined search terms such as: date, postcode, age, and symptoms—psychos*; psychot*, delusion*, voices, and hallucinat*. This returned a set of patient records, which were individually screened by a team of researchers (SO, FB and CGA) using the Psychosis Screening Schedule (PSS) [23]. Researchers swapped cases and rescreened for inter-rater reliability. A 87.4% agreement, along with Kappa score of 0.78, (*p* < 0.01), was achieved. Discrepant or ambiguous cases were resolved by consensus with the principal investigator (CM).

### Inclusion/exclusion criteria

Inclusion criteria for both studies were: resident in the London boroughs of Lambeth or Southwark, (b) aged 18–64 years (inclusive), (c) any psychotic disorder (i.e. ICD F20-29, F30-33) and (d) first contact with mental health services for psychosis. Exclusion criteria were: (1) evidence of psychotic symptoms with an organic cause, (2) transient psychotic symptoms resulting from acute intoxication, and (3) previous contact with services for psychotic symptoms.

We restricted our analyses of both samples to those groups on which we had sufficient numbers for robust estimates of compulsory admissions, i.e. white British, black African black Caribbean and white Other ethnic minority groups.

### Data

For the AESOP sample, patients self-assigned ethnicity. Where this information was not available, other sources such as informants and case notes were used [7]. For the CRIS-FEP sample, self-ascribed ethnicity is collected as part of routine clinical information and this is usually recorded in clinical records. Where this information was missing, ethnicity was ascribed independently by researchers using all available information from the clinical records, including country of birth, nationality, language spoken at home and parents’ country of birth as recommended by the Office for National Statistics [24]. A high inter-rater reliability was achieved between three researchers, who independently extracted and rated ethnicity information on 89 cases blind to each other’s ratings (kappa score = 0.87, *p* < 0.001).

The primary outcome in this study (i.e. compulsory admission) was defined as detention under the UK Mental Health Act (MHA) [25] for assessment and/or treatment on the day of first presentation to mental health services for psychosis. Admissions under the MHA are accurately recorded within CRIS, since it is a statutory clinical data [25, 26]. We coded compulsory admission as a binary outcome (‘yes’ or ‘no’).

Data were collected on demographic and socioeconomic variables using the Medical Research Council Socio-demographic schedule MRC-SDS [27]. Data relating to compulsory admission were collected using a modified form of the Personal and Psychiatric History Schedule (PPHS) [28] for the purpose of data collection from case notes.

### Statistical analysis

Data were analysed using Stata version 12 [29]. First, Chi squared tests were used to compare ethnic groups in each study sample according to the key study variables. Second, we used logistic regression to assess the crude associations between compulsory admission and the key study variables, by study sample. Third, we repeated the logistic regression analyses, this time to test the associations between compulsory admission and ethnicity with complete data. Then, we adjusted for our a priori confounders (age, gender, education qualification, and employment status). Finally, we fitted an interaction term (ethnicity x gender) to our multivariable analyses, and tested whether there were differences between the models using the likelihood-ratio (LR) test. Then, we appended the CRIS-FEP and AESOP samples to assess whether there were differences in compulsory admission by study time points (i.e. AESOP vs. CRIS-FEP). Further, we tested whether compulsory admission outcome differed among those with missing data, particularly in the CRIS-FEP sample, using Chi squared tests.

### Ethical approvals

For the AESOP study, ethical approval was provided in all the sites. The CRIS system was approved as an anonymised dataset for secondary analysis by the Oxfordshire Research Ethics Committee (reference 08/H0606/71). Local approval for this study was obtained from the CRIS Oversight Committee at the BRC South London and Maudsley NHS Foundation Trust (reference: 09–041).

## Results

In the London site of the AESOP study, 266 patients of white British, black African, black Caribbean, or white Other ethnicity with a first episode psychosis and presenting for the first time to services were identified, (mean age 31.9 years, SD 10.3). From the CRIS-FEP study, 446 patients from one of the four ethnic groups were identified, with a mean age 33.6 years (SD 10.8). Table 1 shows the basic characteristics of both AESOP and CRIS-FEP samples.

**Table 1:** Basic characteristics of AESOP and CRIS-FEP samples

	AESOP, *n* = 266 (%)	CRIS-FEP, *n* = 446 (%)
Mean age (sd) years	31.9 (10.3)	33.6 (10.8)
Gender		
Men	112 (56.8)	227 (50.9)
Women	115 (43.2)	219 (49.1)
Ethnicity		
White British	82 (30.8)	133 (29.8)
Black African	61 (22.9)	147 (33.0)
Black Caribbean	97 (36.5)	91 (20.4)
White Other	26 (9.8)	75 (16.8)
Education^a^		
School, no GCSE	87 (34.1)	79 (25.2)
School, GCSE	58 (22.8)	59 (18.9)
Further	72 (28.2)	89 (28.4)
Higher/university	38 (14.9)	86 (27.5)
Employment^b^		
Unemployed	169 (64.5)	275 (67.9)
Student/other	27 (10.3)	47 (11.6)
Employed	66 (25.2)	83 (20.5)
Relationship status^c^		
Single	156 (62.9)	265 (61.9)
Married/in steady relationship	61 (24.6)	103 (24.1)
Divorced/widowed	31 (12.5)	60 (14.0)
Living arrangements^d^		
Alone	134 (50.7)	132 (30.5)
Family/relatives	114 (43.2)	257 (59.3)
Other	16 (6.1)	44 (10.2)
Accommodation arrangements^e^		
Owned	40 (16.8)	53 (19.2)
Rented	180 (75.6)	178 (64.5)
Other (e.g. homeless or refugee hostel)	18 (7.6)	45 (16.3)
Compulsory admission		
No	156 (58.6)	340 (76.2)
Yes	110 (41.4)	106 (23.8)

Missing data

^a^AESOP, 11; CRIS-FEP, 133

^b^AESOP, 4; CRIS-FEP, 41

^c^AESOP, 18; CRIS-FEP, 18

^d^AESOP, 2; CRIS-FEP, 13

^e^AESOP, 28; CRIS-FEP, 170

Compared with AESOP, black Caribbean patients comprised a smaller and black African patients a larger proportion of the CRIS-FEP total sample (Table 1). Patients in the CRIS-FEP study were more likely to be educated to university level, live with family, and to have housing tenure of ‘Other’, i.e. refugee/homeless hostel, than patients in the AESOP study. The proportion of patients in the CRIS-FEP sample (23.8%) compulsorily admitted was notably lower than observed in AESOP (41.4%).

### Associations between ethnicity, compulsory admission and key study variables

We found some ethnic differences by gender in the CRIS-FEP sample, i.e. there were more women in the black Caribbean group (57.1%) than in the white British (48.9%), black African (46.3%) or white Other (45.3%) (Table 2). Whilst educational qualifications appear to have improved between the two samples, a higher proportion of the black Caribbean patients in both samples (42.7% vs. 36.1%) were without secondary school qualifications. Furthermore, employment was lowest among the black African and black Caribbean group in both samples (AESOP: 21.9% and 21.9%; CRIS-FEP: 20.2% and 9.8%, respectively). In the AESOP sample, black African and black Caribbean patients were more likely to live alone (59.0% and 58.3%, respectively), compared with white British patients (37.8%) (Table 2). Furthermore, black African patients in AESOP were less likely to own their homes (5.4%) compared with white British patients (24.3%). These differences were not observed in the CRIS-FEP sample.

**Table 2:** Sample characteristics by ethnicity

Demographic variables	AESOP, *n* = 266 (%)				*p*	CRIS-FEP, *n* = 446 (%)				*p*
	White British (*n* = 82)	Black African (*n* = 61)	Black Caribbean (*n* = 97)	White Other (*n* = 26)		White British (*n* = 133)	Black African (*n* = 147)	Black Caribbean (*n* = 91)	White Other (*n* = 75)	
Age-band										
18–29	43 (52.4)	34 (55.7)	44 (45.4)	9 (34.6)	0.24	56 (42.1)	67 (45.6)	38 (41.8)	23 (30.7)	0.19
30–64	39 (47.6)	27 (44.3)	53 (54.6)	17 (65.4)		77 (57.9)	80 (54.4)	53 (58.2)	52 (69.3)	
Gender										
Men	51 (62.2)	36 (59.0)	46 (47.4)	18 (69.2)	0.10	68 (51.1)	79 (53.7)	39 (42.9)	41 (54.7)	< **0.01**
Women	31 (37.8)	25 (41.0)	51 (52.6)	8 (30.8)		65 (48.9)	68 (46.3)	52 (57.1)	34 (45.3)	
Education										
School, no GCSE	26 (34.2)	11 (19.0)	41 (42.7)	9 (36.0)	**< 0.01**	27 (28.1)	17 (16.5)	22 (36.1)	13 (24.5)	0.06
School, GCSE	13 (17.1)	17 (29.3)	26 (27.1)	2 (8.0)		18 (18.8)	29 (28.2)	8 (13.1)	4 (7.5)	
Further	17 (22.4)	23 (39.7)	24 (25.0)	8 (32.0)		20 (20.8)	29 (28.2)	22 (36.1)	18 (34.0)	
Higher/university	20 (26.3)	7 (12.1)	5 (5.2)	6 (24.0)		31 (32.3)	28 (27.1)	9 (14.7)	18 (34.0)	
Employment										
Unemployed	43 (53.7)	41 (67.2)	67 (69.8)	18 (72.0)	0.06	78 (63.9)	84 (65.1)	66 (80.4)	47 (65.3)	0.06
Student/other	8 (10.0)	10 (16.4)	8 (8.3)	1 (4.0)		15 (12.3)	19 (14.7)	8 (9.8)	5 (6.9)	
Employed	29 (36.3)	21 (21.9)	21 (21.9)	6 (24.0)		29 (23.8)	26 (20.2)	8 (9.8)	20 (27.8)	
Relationship status										
Single	43 (53.1)	32 (58.2)	66 (72.5)	15 (71.4)	0.16	84 (66.1)	82 (85.2)	54 (62.8)	45 (60.8)	0.33
Married/in steady relationship	26 (32.1)	15 (27.3)	15 (16.5)	5 (23.8)		32 (25.2)	32 (22.7)	19 (22.1)	20 (27.0)	
Divorced/widowed	12 (14.8)	8 (15.5)	10 (11.0)	1 (4.8)		11 (8.6)	27 (19.1)	13 (15.1)	9 (12.2)	
Living arrangements										
Alone	31 (37.8)	36 (59.0)	56 (58.3)	11 (44.0)	< **0.01**	41 (32.0)	39 (27.1)	31 (35.2)	21 (28.8)	0.62
Family/relatives	44 (53.7)	19 (31.2)	40 (41.7)	11 (44.0)		78 (61.0)	88 (61.1)	49 (55.7)	42 (57.5)	
Other	7 (8.5)	6 (9.8)	0	3 (12.0)		9 (7.0)	17 (11.8)	8 (9.1)	10 (13.7)	
Accommodation arrangements										
Owned	18 (24.3)	3 (5.4)	16 (18.2)	3 (14.3)	< **0.01**	21 (25.0)	14 (16.1)	9 (18.0)	9 (16.4)	0.42
Rented	53 (71.6)	42 (76.4)	70 (79.5)	15 (71.4)		54 (64.3)	59 (67.8)	32 (64.0)	33 (60.0)	
Other	3 (4.1)	10 (18.2)	2 (2.3)	3 (14.3)		9 (10.7)	14 (16.1)	9 (18.0)	13 (23.6)	
Compulsory admission										
No	62 (75.6)	28 (45.9)	48 (49.5)	18 (69.2)	< **0.01**	110 (82.7)	97 (66.0)	72 (79.1)	61 (81.3)	< **0.01**
Yes	20 (24.4)	33 (54.1)	49 (50.1)	8 (30.8)		23 (17.3)	50 (34.0)	19 (20.9)	14 (18.7)	

The bold values indicate statistically significant results

In both samples, black African (AESOP: 54.1%; CRIS-FEP: 34.0%) and black Caribbean (AESOP: 50.1%; CRIS-FEP: 20.9%) patients were more likely to be compulsorily admitted compared with white British (AESOP: 24.4%; CRIS-FEP: 17.3%) (Table 3).

**Table 3:** Unadjusted odds ratios of compulsory admission and study variables by study sample

Demographic variables	AESOP, *n* = 266				CRIS-FEP, *n* = 446			
	Non-compulsory admission (*n* = 156) (%)	Compulsory admission (*n* = 110) (%)	OR	95% CI	Non-compulsory admission (*n* = 340) (%)	Compulsory admission (*n* = 106) (%)	OR	95% CI
Age-band								
18–29	80 (61.5)	50 (38.5)	1.00		140 (76.1)	44 (23.9)	1.00	
30–64	76 (58.9)	60 (44.1)	1.26	0.77–2.06	200 (76.3)	62 (23.7)	0.98	0.63–1.53
Gender								
Men	91 (60.3)	60 (39.7)	1.00		165 (72.7)	62 (27.3)	1.00	
Women	65 (56.5)	50 (43.5)	1.16	0.71–1.90	175 (79.9)	44 (20.1)	0.66	0.43–1.04
Ethnicity								
White British	62 (75.6)	20 (24.4)	1.00		110 (82.7)	23 (17.3)	1.00	
Black African	28 (45.9)	33 (54.1)	**3.16**	1.66–6.01**	97 (66.0)	50 (34.0)	**2.46**	1.40–4.33**
Black Caribbean	48 (49.5)	49 (50.5)	**3.65**	1.79–7.44**	72 (79.1)	19 (20.9)	1.26	0.64–2.48
White Other	18 (69.2)	8 (30.8)	1.37	0.52–3.64	61 (81.3)	14 (18.7)	1.09	0.52–2.28
Education								
School, no GCSE	50 (57.5)	37 (42.5)	0.91	0.42–1.96	60 (75.9)	19 (24.1)	0.81	0.40–1.64
School, GCSE	33 (56.9)	25 (43.1)	0.93	0.41–2.13	45 (76.3)	14 (23.7)	0.80	0.37–1.72
Further	43 (59.7)	29 (40.3)	0.83	0.37–1.84	67 (75.3)	22 (24.7)	0.84	0.43–1.66
Higher/university	21 (55.3)	17 (44.7)	1.00		62 (72.1)	24 (27.9)	1.00	
Employment								
Unemployed	88 (52.1)	81 (47.9)	**2.65**	1.41–4.97**	213 (77.4)	62 (22.6)	0.85	0.48–1.51
Student/other	17 (63.0)	10 (37.0)	1.69	0.65–4.41	34 (72.3)	13 (27.7)	1.12	0.50–2.53
Employed	49 (74.2)	17 (25.8)	1.00		62 (74.7)	21 (25.3)	1.00	
Relationship status^3^								
Single	93 (59.6)	63 (40.4)	0.85	0.46–1.55	197 (74.3)	68 (25.7)	1.43	0.81–2.50
Divorced/widowed	18 (58.1)	13 (41.9)	0.90	0.37–2.17	47 (78.3)	13 (21.7)	1.14	0.52–2.51
Married/in steady relationship	34 (55.7)	27 (44.3)	1.00		83 (80.6)	20 (19.4)	1.00	
Living arrangements								
Family/relatives	79 (69.3)	35 (30.7)	1.00		199 (77.4)	58 (22.6)	1.00	
Alone	68 (50.7)	66 (49.2)	**2.19**	1.29–3.69**	100 (75.8)	32 (24.2)	1.09	0.66–1.79
Other	9 (56.3)	7 (43.7)	1.75	0.60–5.09	32 (72.4)	12 (27.3)	1.28	0.62–2.65
Accommodation arrangements								
Owned	25 (62.5)	15 (37.5)	1.00		39 (73.6)	14 (26.4)	1.00	
Rented	102 (56.7)	78 (43.3)	1.27	0.62–2.57	136 (76.4)	42 (23.6)	0.86	0.42–1.73
Other	8 (44.4)	10 (55.6)	2.08	0.67–6.44	31 (68.9)	14 (31.1)	1.25	0.52–3.02

The bold values indicate statistically significant results

*p ≤ 0.05; **p ≤ 0.01

In the AESOP sample, patients who were compulsorily admitted were more likely to be unemployed (OR = 2.65; 95%CI = 1.41–4.97) and live alone (OR = 2.19; 95% CI = 1.29–3.69). (Table 3). In the CRIS-FEP sample, there was weak evidence of a lower likelihood of compulsory admission among women (OR = 0.66; 95% CI = 0.43–1.04). No further differences were observed in both samples.

### Associations between compulsory admission and ethnicity

In analyses of ethnicity and compulsory admission, first, we estimated unadjusted odds ratios in the full sample, which showed that both black African and black Caribbean patients were three times more likely to be detained in the AESOP sample (OR = 3.16; 95% CI = 1.66–6.01 and OR = 3.65; 95% CI = 1.79–7.44), respectively. In the CRIS-FEP sample, black African ethnicity was strongly associated with compulsory admission (OR = 2.46; 95% CI = 1.40–4.33), for the black Caribbean patients there was no evidence of an association (OR = 1.26; 95% CI = 0.64–2.48) (Table 3). Then, we restricted the samples to those for whom data were complete on the variables included in the multivariable regression models, i.e. AESOP, *n* = 253 and CRIS-FEP, *n* = 291. We found that the unadjusted association between ethnicity and compulsory admission was held. In both samples (Model 1, Table 4), black African patients (AESOP: OR = 3.89, 95% CI = 1.86–8.11; CRIS-FEP: OR = 3.11, 95% CI = 1.52–6.32) were around three times more likely than white British to be compulsorily admitted. In the AESOP sample, black Caribbean patients (AESOP: OR = 3.01; 95% CI = 1.55–5.81) were similarly around three times more likely to be compulsorily admitted. However, in CRIS-FEP, there was no evidence of association (OR = 1.53; 95% CI = 0.66–3.51). When we adjusted for our a priori confounders (i.e. demographic and socioeconomic variables), these findings remained, with odds ratios largely unchanged. (Model 2, Table 4).

**Table 4:** Unadjusted and adjusted odds ratios of associations between ethnicity and compulsory admission

	AESOP, *n* = 253 odds ratios (95% CI)			CRIS-FEP, *n* = 291 odds ratios (95% CI)		
	Model 1	Model 2	Model 3^a^	Model 1	Model 2	Model 3^b^
Ethnicity						
White British	1.00	1.00	1.00	1.00	1.00	1.00
Black African	3.89 (1.86 − 8.11)**	3.96 (1.80 − 8.71)**	6.27 (0.68–57.22)	3.11 (1.55–6.23)**	3.12 (1.52–6.35)**	0.75 (0.12–4.41)
Black Caribbean	3.01 (1.55 − 5.81)**	3.20 (1.56 − 6.54)**	2.32 (0.28–19.12)	1.53 (0.66–3.51)	1.68 (0.71–3.98)	0.39 (0.05–2.58)
White Other	1.14 (0.41–3.16)	1.00 (0.35–2.87)	1.07 (0.27–4.17)	1.06 (0.42–2.62)	1.01 (0.40–2.55)	0.50 (0.14–1.79)

The bold values indicate statistically significant results

Model 3—adjusted for age, gender, employment status and education qualification, plus interaction term (ethnicity × gender)

Model 2—adjusted for age, gender, employment status and education qualification

Model 1—unadjusted

*CI* confidence intervals

^a^Likelihood test between main effects (Model 2) vs. with interaction term (Model 3): *X*^2^ = 4.74, *p* = 0.19

^b^Likelihood test between main effects (Model 2) vs. with interaction term (Model 3): *X*^2^ = 3.24, *p* = 0.35

*p ≤ 0.05 **p ≤ 0.01

When we fitted an interaction term in the final Model 3, (Table 4), the odds of compulsory admission was attenuated for black Caribbean (in both samples), black African and white Other patients (in CRIS-FEP). Overall, we did not find evidence of effect modifications between the main effects and interaction term for ethnicity and compulsory admission in the AESOP sample (LR test *X*^2^ = 4.74, *p* = 0.19) or the CRIS-FEP sample (LR test *X*^2^ = 3.24, *p* = 0.35). In analyses of study time point (AESOP vs. CRIS-FEP) and compulsory admission, we found that the CRIS-FEP sample was less likely to compulsorily detained (*n* = 544), OR = 0.72; 95% CI = 0.61–0.84 and adj. OR = 0.71; 95% CI = 0.60–0.85 compared with the AESOP sample. Finally, when we tested for differences in compulsory admission outcome among individuals with missing data, chiefly, educational qualifications (detained vs. non-detained:  25.5% vs. 31.1%, *X*^2^ 1.74, *p* = 0.78) and employment status (detained vs. non-detained:  9.4% vs. 9.1%, *X*^2^ 0.73, *p* = 0.86), there were no differences.

## Discussion

### Main findings

In this population-based study of compulsory admission at first presentation for psychosis in south London, UK, we found, contrary to our hypothesis, that the odds of compulsory admission by ethnicity have remained largely unchanged. The most striking findings were in black African patients, for whom the odds of compulsory admission remained three times higher, compared with white British patients, between the two time periods, from 1997 to 1999 and from 2010 to 2012. We found insufficient evidence of reduced odds of compulsory admission for black Caribbean patients compared with findings of 15 years ago. However, we found that overall the proportions of compulsory admissions have gone down, for black African (54.1% vs. 34%), black Caribbean (50.1% vs. 20.9%), white Other (30.8% vs. 18.7%) and white British (24.4% vs. 17.3%) patients, between the two time periods in this study. There was no evidence of effect modifications between the main effects only and with interaction term for ethnicity and compulsory admission across the AESOP and CRIS-FEP samples. However, we found that CRIS-FEP patients were less likely to be compulsorily detained.

### Methodological consideration

The key strengths of this study are its large sample size and the comparison of two datasets collected in the same catchment area at different time points. These allowed the investigation of ethnic differences in compulsory admission during FEP at two different time points in the UK. Our large sample sizes also enabled us to control for several factors that may explain the higher rates of compulsory admission. Our study adds to the steadily growing number of large epidemiological studies with sufficient power to investigate the issue of compulsory admissions among black Caribbean and black African patients as separate ethnic groups. In addition, we applied traditional epidemiological research methods used in the AESOP study to the CRIS electronic health records and we successfully identified FEP cases within the CRIS system.

While our study has a number of advantages over previous studies, there are still a number of limitations. The missing data, particularly in the multivariable analyses may have affected our results. Further, our results should be treated with caution due to the wide confidence intervals particularly for the black Caribbean patients. The use of multiple imputations could have provided a more complete estimation of the odds of compulsory admission particularly for this group. However, given that the missing values for the educational qualifications and employments status variables did not differ by compulsory admissions outcome, the complete case analyses that we employed were considered appropriate. It is also noteworthy that the CRIS clinical data are recorded by clinicians for clinical purposes and not collected for research; therefore, the accuracy of information depends on the quality of clinicians’ documentation, particularly socioeconomic information. However, given that our outcome data (compulsory admission) are statutory data, it is unlikely that anyone admitted under the Mental Health Act would have not been recorded in the electronic health records/CRIS. In addition, given this is a single-centre study, our findings may not be generalizable to other areas.

A key methodological issue in epidemiological research is selection bias. The database used in the CRIS-FEP study is representative of the population of South London. This is because SLaM is the only mental healthcare provider for our study catchment area, and people presenting with major mental health issues such as psychosis tend to present to specialist mental health services directly or are referred by their general practitioners or emergency department to specialist mental health services, as private sector provision is minimal. This means that psychosis cases and compulsory admission data in our study can be considered complete of case ascertainment, and the likelihood of bias is minimal.

### Relationship to previous studies

Our findings are consistent with previous studies [8, 13, 18, 30]. Mann and colleagues (2014) found in a sample of 674 patients that black African patients had threefold increased odds of being compulsorily admitted at first contact, but they did not find this association in black Caribbean patients. Similarly, Lawlor and colleagues (2012) in a sample of 287 women admitted to inpatient and crisis services found higher rates of compulsory admission among black African patients. Evidence from the rest of Europe also suggests a similar trend. For example, in a Dutch cohort study of psychotic patients, Van der Post et al. (2012), having adjusted for a sociodemographic confounders, also replicated UK findings that sub-Saharan African patients were more likely to be detained compulsorily (adj. OR = 3.0; 95% CI = 1.4–6.4). Another Dutch study showed that Surinamese patients were three times more likely to be compulsorily detained compared with the native Dutch patients [31]. Our finding of no association between black Caribbean ethnicity and compulsory admission are in keeping with previous research [32–34]. In addition, consistent with previous studies was our finding of no difference in compulsory admission between white British and white Other patients [13]. Adjusting for socioeconomic status as a confounder has been argued as important for studies investigating the relationship between ethnicity and mental health [35]. We were able to do so in this study. In contrast to some previous studies that reported associations between men of black Caribbean and black African ethnicity with compulsory admission [7, 13], we did not find evidence to support this.

### Explaining the difference

Making sense of the striking ethnic differences in compulsory admission in this study may be considered in three key ways. First, over a few decades, the way UK mental health services are structured and provided has changed considerably. Hospital beds have been reduced significantly and there has been a shift towards community-based treatment [3, 36]. A number of community-based and crisis resolution initiatives such as early intervention for psychosis, home treatment and outreach services were introduced in the early 2000s, with a view to managing patients that would normally require acute hospital admission, at home [4]. These services are responsible for assessing, treating and supporting people in psychiatric emergencies in their own homes and are available 24 h a day. Consequently, the focus on hospital admissions is on prevention and speedy discharge. For a patient to be admitted to hospital, they have to be extremely unwell [4], possibly posing danger to themselves and others, and, therefore, requiring compulsory admission. A number of these new services were not operational during the period the AESOP study was conducted. This may explain the overall reduction in the rate of compulsory admissions between the two study periods. It may also explain the higher rates of admission and compulsory admission among both black African and black Caribbean patients in the AESOP sample. For example, the introduction of early intervention psychosis was anticipated to make a significant contribution to earlier treatment, less coercive care and so improve outcomes [5].

Second, social disadvantage factors, which have been linked to the higher rate of psychosis and coercive pathways to care during FEP [37–39] are important markers of health inequalities in our society [40, 41]. For example, our data show that compared with 15 years ago, the proportion of black Caribbean patients who were unemployed rose from 69.8% (AESOP) to 80.4% (CRIS-FEP). The increase in unemployment between the two samples may reflect the UK economic landscape following the global financial crisis in 2008, during which youth employment declined and those at the margins of the society were hit the hardest [42]. Similarly, the proportion of patients with insecure living arrangements such as homeless hostel also rose for black African, 9.8% (AESOP) to 11.8% (CRIS-FEP) and black Caribbean, 0% (AESOP) to 9.1% (CRIS-FEP). Although, both unemployment and living circumstances were associated with compulsory admission in the AESOP sample, they may explain only a proportion of the ethnic variations. Furthermore, our data suggest that the proportion of people in relationship or living with others compared with 15 years ago have improved. For example, although we did not specifically measure generational status in our study, given that black Caribbean people have mostly settled in the UK post-World War II [1, 38], it is possible that there may have been a few generations of black Caribbean people, as noted in the wider UK population [43]. Therefore, it is plausible that the black Caribbean group may have developed cumulative community social networks that encourage mutual support, a sense of belonging and enriched social relationships through social capital and acculturation [44, 45]. The absence of these factors has been well documented as post-migratory risk factors of psychosis [41, 46, 47]. Such risks may still be present for the black African population, whose integration into the UK society may not be as established as those of the black Caribbean ethnic group.

Third, we found no evidence of an association between compulsory admissions and white Other patients in both AESOP and CRIS-FEP samples. While this finding is consistent with previous studies [8, 13, 48], it is intriguing, given the arrival of a large number of white migrants from Eastern Europe into the UK since the expansion of the EU in 2004 [2]. During the same periods, black African people were also arriving in the UK, resulting in around a 2.4% increase in the black African population in London [49]. We found the most striking odds of being compulsorily detained in the black African ethnic group. This is important, black African patients may perceive seeking help from mental health services as stigmatising, unfair, and discriminatory, as has been reported in previous studies [50–52] and so they may be reluctant to come into hospital voluntarily. However, the association of compulsory admission with black African and black Caribbean ethnicity also draws attention to the social structures and processes, including institutional discrimination that shape access to material resources and health services, which in turn contribute to differences in health outcomes. Our findings of involuntary admission among black African patients are consistent with findings of wider inequalities in many marginalised and disadvantage groups [53]. By contrast, we found no differences in the odds of compulsory admission between the white ‘Other’ and white British patients. This may be explained from immigration regulations and socioeconomic circumstances perspective. For example, it is possible that the white ‘Other’ (mainly European) population in the UK experience less stressful acculturation process and lower perceived discrimination [54], since they have full legal entitlement to free health care and the labour market [55] compared with other migrants from elsewhere who may experience insecure immigration status, e.g. asylum seekers or refugees. Although not measured in our study, we speculate that, given the potential differences in the aforementioned immigration status between non-UK-born people of black African and white ‘Other’ ethnicity, it could be argued that the potential insecurities faced by those who migrated to the UK from outside of Europe may contribute to poorer health outcomes [55].

There are further nuances in our data that are noteworthy. For example, the data hint at the disparity between white British and black Caribbean patients may be narrowing as the proportions of compulsory detention between the two groups are now relatively similar (17.3% vs. 20.9%, respectively). This may suggest that the factors that contributed to differences in previously reported findings such as a lack of understanding of health services, distrust in mental health services and reduced family involvement [50, 51] may no longer be as relevant to the black Caribbean groups. It is also possible that black Caribbean people are more confident in navigating the healthcare services when seeking help and so may be persuaded to come involuntarily.

## Conclusions

Our results show that ethnicity remains a strong predictor of compulsory admission, with black African patients mostly at risk. Whilst our findings hint at small improvements in the pathways to care, they also highlight that more research is needed to further shine light on the factors which influence being detained compulsorily in both black African and black Caribbean patients. At its heart, the Independent Review of the Mental Health Act, seeks (among others) “to make the MHA work better for everyone”, “reduce disparities between groups with protected characteristics” [17]. Our findings provide evidence that may be used to inform the review in making recommendations for effective mental health services for people from ethnic minority groups.
